# Supplementary material for: Determinants of raffinose family oligosaccharide use in Bacteroides species
Source: J Bacteriol. 2024 Sep 27;206(10):e00235-24. doi: 10.1128/jb.00235-24 (PMC11501099; doi:10.1128/jb.00235-24)
Supplement: Supplemental figures and legends — Fig. S1-S8. [file jb.00235-24-s0001.docx]

**Supplementary Figures**

**
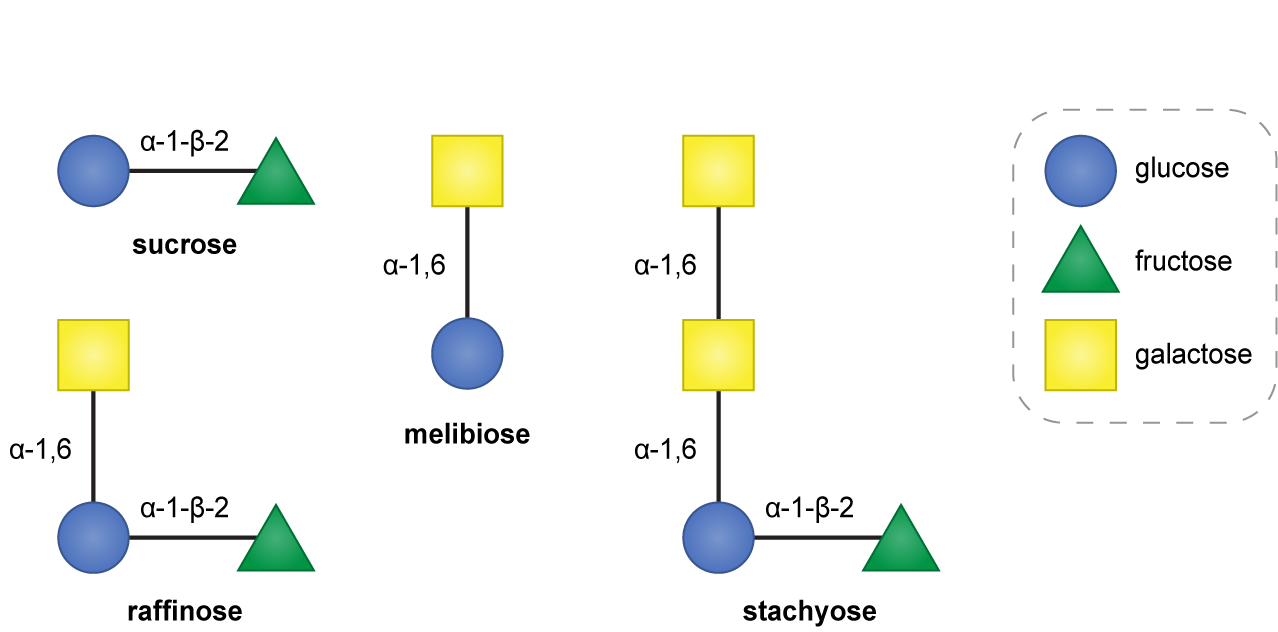
**

**Figure S1. General structure of Raffinose Family Oligosaccharides (RFOs).**

RFOs are represented with subunits and their linkages indicated next to them. Sucrose and melibiose are disaccharide subunits of RFOs. The monosaccharide subunits which make up RFOs are indicated in the box.

**Figure S2. *BT1871* is the only gene in PUL24 important for RFO utilization in *B. thetaiotaomicron***

(A) Growth curves of WT dupl-, *BT1871* mutant and *BT1871* *BT1876* double mutant strains on RFOs. Points and error bars represent the mean and SD of n=3 biological replicates. On AMG, both the Δ*BT1871* strain and the double mutant failed to grow. On melibiose, the Δ*BT1871* strain had a mean growth rate of 0.12 h^-1^ for the compared to 0.09 h^-1^ for the double mutant (p=0.26 by two tailed t-test) and on raffinose the Δ*BT1871* strain had a mean growth rate of 0.08 h^-1^ compared to 0.09 h^-1^ for the double mutant (p=0.23 by two-tailed t-test).

(B) One representative growth curve from an assay to find mutants capable of growth on AMG as in Fig. 2C. Growth curves from individual wells are depicted.

For each growth curve, the sugar used as the sole carbon source is indicated at the top along with the concentration used.

(

**B**

**A**

**A**

**B**

**Figure S3. The ⍺-galactosidases *BT2851* and *BT3131,* the dehydrogenase *BT2158,* and the glycosidase *BT2157* are not involved in RFO utilization in *B. thetaiotaomicron.***

(A) Growth curves of WT, *BT3131,* and *BT2851* mutant strains on RFOs. On melibiose the WT strain had a mean growth rate of 0.22 h^-1^, compared to 0.16 h^-1^ for the Δ*BT2851* strain and 0.20 h^-1^ for the Δ*BT3131* strain (p>0.05 for each comparison of a mutant strain to the WT strain using two-tailed t-test). On raffinose, the WT strain had a mean growth rate of 0.20 h^-1^, compared to 0.16 h^-1^ for the Δ*BT2851* strain and 0.17 h^-1^ for the Δ*BT3131* strain (p>0.05 for each comparison of a mutant strain to the WT strain using two-tailed t-test. On stachyose, the WT strain had a mean growth rate of 0.16 h^-1^, compared to 0.12 h^-1^ for the Δ*BT2851* strain and 0.14 h^-1^ for the Δ*BT3131* strain (p>0.05 for each comparison of a mutant strain to the WT strain using two-tailed t-test.

(B) Growth curves of WT, *BT2157,* and *BT2158* mutant strains on RFOs. (D) Growth curves of WT, *BT1871*, *BT1871 BT2157*, *BT1871 BT2158* mutants on RFOs. On raffinose mean growth rate of the WT strain was 0.20 h^-1^, compared to 0.21 h^-1^ for the Δ*BT2157* strain and 0.17 h^-1^ for the Δ*BT2158* strain (p>0.05 for each comparison of a mutant strain to the WT strain using two-tailed t-test). On stachyose the mean growth rate of the WT strain was 0.15 h^-1^, compared to 0.14 h^-1^ for the Δ*BT2157* strain and 0.13 h^-1^ for the Δ*BT2158* strain, p>0.05 for each comparison of a mutant strain to the WT strain using two-tailed t-test.

In all panels, points and error bars represent the mean and SD of n=3 biological replicates. For each growth curve, the sugar used as the sole carbon source is indicated at the top along with the concentration used.

**Figure S4. Growth of *B. thetaiotaomicron* on raffinose as the sole carbon source leads to upregulation of PUL22 genes compared to growth on glucose.**

Volcano plot depicting differentially expressed genes on raffinose compared to glucose. The dashed black lines indicate the threshold used to determine significance (absolute log 2 fold change > 1 and adjusted p value < 0.05). Top 10 highest differentially expressed genes are labeled. The black box highlights PUL22 genes which are highly upregulated on raffinose.

*BT1757* - putative fructokinase, *BT1758* – putative inner membrane fructose transporter, *BT1759* – GH32 family sucrase, *BT1760* – GH32 family sucrase, *BT1761* – SusE-like gene, *BT1762* – SusD-like gene, *BT1763* – SusC-like TonB dependent transporter, *BT1765* – GH32 family sucrase.

**A**


**B**


**Figure S5. Locations of novel 5' ends found in PUL24 using 5' RACE**

(A) and (B) represent the location of novel 5' ends for *BT1875* and *BT1871* respectively, annotated using 5' RACE as depicted in Fig. 5C. The identified ends are shaded blue with a bent arrow on top. The start and stop codons of genes are shaded red.

**A**

**Figure S6. The PUL24 sigma factor *BT1877* is required for better growth of *BT1876* mutants on RFOs.**

(A) Growth curves of WT and *BT1877* (PUL24 sigma factor) mutant strain on RFOs. (B) Growth curves of WT, *BT1876* (PUL24 anti-sigma factor) and *BT1876 BT1877* double mutant strains on RFOs. In all panels, points and error bars represent the mean and SD of n=3 biological replicates. For each growth curve, the sugar used as the sole carbon source is indicated at the top along with the concentration used. On melibiose the mean growth rate of the WT strain was 0.20 h^-1^ compared to 0.29 h^-1^ for the Δ*BT1877* strain (p=0.78 by two-tailed t-test). On raffinose the mean growth rate of the WT strain was 0.12 h^-1^ compared to 0.11 h^-1^ for the Δ*BT1877* strain (p=0.16 by two-tailed t-test). On stachyose the *BT1877* mutant grew slightly better than the WT (mean growth rate of the WT strain was 0.06 h^-1^ compared to. 0.08 h^-1^ for the Δ*BT1877* strain, p=0.0047 by two-tailed t-test).

**B**

**B**

**A**

**Figure S7. Differences in maximum ODs and growth rates on raffinose for *Bacteroides* species with or without a *BT1871* homolog.**

Boxplots showing comparison of maximum OD630 values (A) and growth rate (B) of *Bacteroides* species either with or without a *BT1871* homologue. Individual values were taken from growth curves depicted in 6A and 6B.

Comparisons were done using a Mann Whitney U test and was found to be significant *, P<0.05 for (A) but not for (B).

**Figure S8. The constitutive *BT1871* expression cassette is active and restores growth on RFOs in a *BT1871* mutant strain.**

Growth curves of WT, *BT1871* mutant, and a *BT1871* mutant strain constitutively expressing *BT1871* under the control of the housekeeping sigma rpoD promoter (ΔBT1871:pNBU2_BT1871) on RFOs. Points and error bars represent the mean and SD of n=3 biological replicates. For each growth curve, the sugar used as the sole carbon source is indicated at the top along with the concentration used.
